# Real-time cell toxicity profiling of Tox21 10K compounds reveals cytotoxicity dependent toxicity pathway linkage

**DOI:** 10.1371/journal.pone.0177902

**Published:** 2017-05-22

**Authors:** Jui-Hua Hsieh, Ruili Huang, Ja-An Lin, Alexander Sedykh, Jinghua Zhao, Raymond R. Tice, Richard S. Paules, Menghang Xia, Scott S. Auerbach

**Affiliations:** 1 Kelly Government Solutions, Durham, North Carolina, United States of America; 2 National Center for Advancing Translational Sciences, National Institutes of Health, Rockville, Maryland, United States of America; 3 US Food and Drug Administration, Silver Spring, Maryland, United States of America; 4 Sciome, Durham, North Carolina, United States of America; 5 Division of the National Toxicology Program, National Institute of Environmental Health Sciences, National Institutes of Health, Durham, North Carolina, United States of America; University of Louisville School of Medicine, UNITED STATES

## Abstract

Cytotoxicity is a commonly used *in vitro* endpoint for evaluating chemical toxicity. In support of the U.S. Tox21 screening program, the cytotoxicity of ~10K chemicals was interrogated at 0, 8, 16, 24, 32, & 40 hours of exposure in a concentration dependent fashion in two cell lines (HEK293, HepG2) using two multiplexed, real-time assay technologies. One technology measures the metabolic activity of cells (i.e., cell viability, *glo*) while the other evaluates cell membrane integrity (i.e., cell death, *flor*). Using *glo* technology, more actives and greater temporal variations were seen in HEK293 cells, while results for the *flor* technology were more similar across the two cell types. Chemicals were grouped into classes based on their cytotoxicity kinetics profiles and these classes were evaluated for their associations with activity in the Tox21 nuclear receptor and stress response pathway assays. Some pathways, such as the activation of H2AX, were associated with the fast-responding cytotoxicity classes, while others, such as activation of TP53, were associated with the slow-responding cytotoxicity classes. By clustering pathways based on their degree of association to the different cytotoxicity kinetics labels, we identified clusters of pathways where active chemicals presented similar kinetics of cytotoxicity. Such linkages could be due to shared underlying biological processes between pathways, for example, activation of H2AX and heat shock factor. Others involving nuclear receptor activity are likely due to shared chemical structures rather than pathway level interactions. Based on the linkage between androgen receptor antagonism and Nrf2 activity, we surmise that a subclass of androgen receptor antagonists cause cytotoxicity via oxidative stress that is associated with Nrf2 activation. In summary, the real-time cytotoxicity screen provides informative chemical cytotoxicity kinetics data related to their cytotoxicity mechanisms, and with our analysis, it is possible to formulate mechanism-based hypotheses on the cytotoxic properties of the tested chemicals.

## Introduction

In the U.S. Tox21 program, a 10K chemical library is being evaluated for toxicological potential using mechanism-based, cell-based quantitative high throughput screening (qHTS) assays (“toxicity pathways”) that focus on nuclear receptor and stress response pathways [1]. As of 2016 August, over 40 assays have been screened and the results released in PubChem (https://www.ncbi.nlm.nih.gov/pcassay/?term=%22tox21%22). The goals of Tox21 include the prioritization of chemicals with little or no toxicological data for a more in-depth toxicological evaluation based on mechanism-based activity data and the development of models for better predicting *in vivo* toxicity. For example, data from a battery of Tox21 estrogen receptor (ER) related assays have been used in building a model for predicting ER dependent endocrine disruption potential [2,3]. In addition, *in vitro* to *in vivo* extrapolation (IVIVE) analysis based on the Tox21 screening data is being conducted to predict the likelihood of activity in exposed humans [4]. However, to date, no large-scale analysis has been conducted to characterize the relationship between chemical-induced cell-based pathway perturbations and the cytotoxicity of the Tox21 10K chemicals. Prioritizing chemicals based on cytotoxicity relevant cell-based pathway perturbations could provide more phenotypically relevant, mechanism-based hypotheses for toxicological testing.

Cell death plays an important role in chemical-induced toxicity in humans [5]. Many different modes of action (MOA) can lead to cytotoxicity and in order to understand the underlying mechanisms, hypotheses need to be generated and evaluated. By interrogating cytotoxicity in a sufficiently large number of cell lines with diverse genetic features, chemicals with similar MOAs can be grouped together based on their differential cytotoxic responses across cell lines [6–9]. One example of this approach is the identification of novel kinase inhibitors based on their cytotoxicity profiles in 102 cancer cell lines by comparing the similarity of their profiles to known kinase inhibitors [9]. In addition to the pattern of cytotoxicity across cell lines, the kinetics of cytotoxicity can vary greatly for different groups of chemicals [10–12]; for example, immediate cellular changes can be seen for chemicals acting on ion channels, while a delayed cytotoxic response occurs for chemicals that act on cell cycle processes. However, it has also been shown that many chemicals with different pharmacological effects can display similar kinetics for cytotoxicity, implying that they share some underlying common mechanisms leading to cell death, despite their seemingly unrelated pharmacological functions [10]. Without some prior assumptions and data, identifying the underlying common mechanisms can be a challenging experimental task.

In this study, chemical-induced cytotoxicity at six different time points (0, 8, 16, 24, 32, & 40 hours) was interrogated in two cell lines, HEK293, a human embryonic kidney cell line, and HepG2, a human hepatocellular carcinoma cell line, using two multiplexed, real-time assay technologies: the Promega RealTime-Glo™ MT Cell Viability Assay and the Promega CellTox™ Green Cytotoxicity Assay. The former measures the reducing potential of cells and thus their metabolic ability (i.e., cell viability) based on a luciferase substrate produced in live cells only while the latter detects the loss of cellular membrane integrity (i.e., cell death) based on a DNA-binding dye preferentially excluded from live cells. Results from the four assays (i.e., two assays each performed in two different cell lines) were compared in terms of the number of actives, kinetics of response, and potency correlation. The active chemicals were then grouped based on their similarity of cytotoxicity profiles (degree, mechanisms, and kinetics of cell death/cell viability). Based on the assumption that groups of chemicals with similar cytotoxicity profiles can have similar MOAs, which may be represented by activities in the Tox21 stress response and nuclear receptor related pathway assays, we identified significant associations between pairs of individual chemical cytotoxicity kinetics grouping and individual pathway. By clustering these pathways based on their degree of association to the different cytotoxicity kinetics labels, we identified clusters of pathways where active chemicals presented similar kinetics of cytotoxicity. We investigated some of the linkages (or clusters) using known receptor antagonists and identified a possible MOA that could account for the observed cytotoxicity effect. The identified relationships could be seen as *in vitro* biological features that could help to differentiate true actives from the assay artifacts in these *in vitro* assays [13,14] and thus, could be used to prioritize the actives identified in previous Tox21 screens. In summary, by linking the phenotypic outcomes in this study with Tox21 cell-based, target-specific data, we can formulate hypotheses as to the mechanisms of cytotoxicity produced by chemicals in the Tox21 library.

## Methods

### Tox21 10K chemical library

The original Tox21 compound library consisted of ~12,500 (~8,300 unique CAS Registry Number (CASRN)) compounds procured from commercial sources by the U.S. Environmental Protection Agency (EPA), the National Institute of Environmental Health Sciences (NIEHS)/National Toxicology Program (NTP), and the National Institutes of Health (NIH) National Center for Advancing Translational Sciences (NCATS)). The library consists of a large variety of chemicals, including pesticides, industrial chemicals, natural food products, and drugs. The latter category includes failed drugs that did not make it to market, drugs that are no longer marketed, and drugs that are marketed currently. With usage, portions of the library have been replaced and for this study, 9,667 compounds (7,872 unique CASRN) were screened. Each substance was prepared as a stock solution (generally at 20 mM) in dimethyl sulfoxide (DMSO) and was serially diluted to yield 15 concentrations generally ranging from 1 nM to 77 μM (final concentrations in the assay wells, concentration spacing ~ 0.35 log_10_ unit, 2.2 fold). Eighty-eight duplicate compounds were intentionally included on each of the 1536-well screening plates to evaluate technical variability across plates and runs. The list of unique compounds, including chemical names and CASRNs, as well as curated chemical structures and structure identifiers (formula, systematic names, SMILES, desalted SMILES, InChI) can be downloaded from the EPA website (https://www3.epa.gov/research/COMPTOX/toxcast_chemical_info.html). The results of the chemical purity analysis can be found in the Tox21 Samples web site (https://tripod.nih.gov/tox21/samples).

### Assays and qHTS

**Fig 1:**
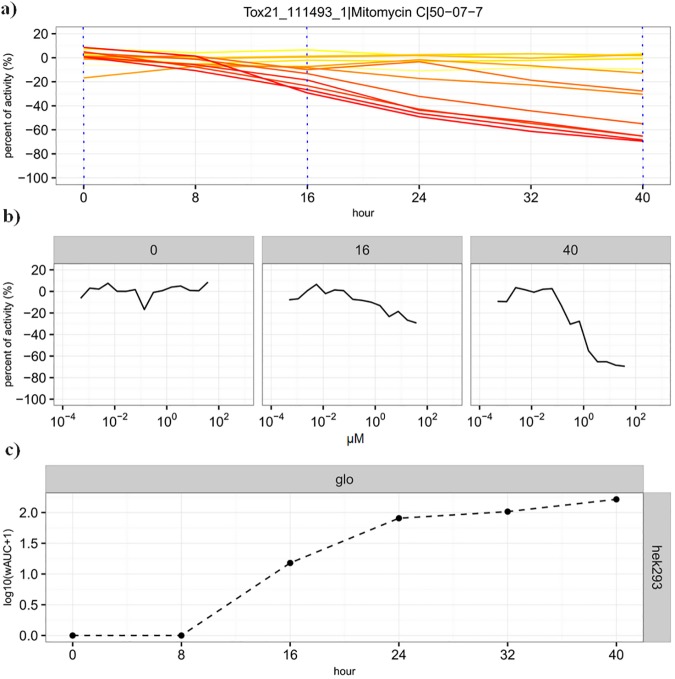
Cytotoxicity kinetics data of mitomycin C in HEK293 cell line using *glo* technology. a) The percent of activity is plotted as the function of hour. The color represents different concentrations of the chemical. The darker color (redder) is equivalent to higher concentrations. b) The concentration-response data at three representative time points (0, 16, 40 hour). The total effect across concentrations can be summarized as wAUC. c) The total effect (log_10_(wAUC+1)) is plotted as the function of exposure duration in hours.

Cell viability was measured using the RealTime-Glo™ MT Cell Viability Assay (Promega, Madison, WI, USA) while cell death was measured using the CellTox™ Green Cytotoxicity Assay (Promega). Both assays are nonlytic and homogeneous. In the former assay (*glo*, in the following text), the number of viable cells is proportional to the amount of luminescent signal, which is directly proportional to the amount of NanoLuc® substrate used in the NanoLuc® luciferase reaction. The NanoLuc® substrate is only produced in live cells where the cell-permeant prosubstrate is reduced [15,16]. Thus, as the number of metabolically active cells decreases, the *glo* signal decreases proportionally. The latter assay (*flor*, in the following text) measures changes in membrane integrity that occur as a result of cell death (i.e., an increase in the number of dead cells is proportional to the increase in fluorescent signal, which results from the increased number of DNA-dye aggregates detected due to the loss of cell membrane integrity) [17]. Using the two real-time assays in a multiplexed mode, we measured in a single run the changes in cell viability and cell death in two cell lines induced by the Tox21 chemicals at 15 concentrations with a single well per concentration in a qHTS format (1536 well) after 0 (right after the chemical administration), 8, 16, 24, 32, and 40 hours of exposure. These sample times were selected for the screening convenience. The two cell lines used in this study were HEK293, a human embryonic kidney line, and HepG2, a human hepatocellular carcinoma cell line. Selection of these two cell lines was based on their extensive use in many other Tox21 reporter assays. In total, >11K concentration-response curves were generated at each time point using a single assay and a single cell line, with >266K concentration-response curves were generated in this study. The plate/well level data can be downloaded from the UNC Dataverse (https://dataverse.unc.edu/dataset.xhtml?persistentId=doi:10.15139/S3/12321) and are available in PubChem (https://www.ncbi.nlm.nih.gov/pcassay?term=tox21+real+time). The cytotoxicity kinetics data of mitomycin C in the HEK293 cell line using the *glo* assay technology (HEK293[*glo*], in the following text) is provided in Fig 1 as an example of data handling.

#### Cell culture

HepG2 and HEK293 cells were purchased from the American Type Culture Collection (ATCC, Manassas, VA, USA). Cells were dispensed at 800 HEK293 or 600 HepG2 cells/6 μL/well in tissue-culture treated 1,536-well white wall/solid bottom assay plates (Greiner Bio-One North America, Monroe, NC, USA) using a Flying Reagent Dispenser (Aurora Discovery, Carlsbad, CA, USA). Both HepG2 and HEK293 cells were incubated at 37°C for 5 hours to allow for cell attachment, followed by addition of compounds via a pin tool station (Kalypsys, San Diego, CA, USA). After compound addition, plates were read using a ViewLux plate reader at 0 (right after addition), 8, 16, 24, 32, & 40 hours. Eight hours is the optimal time interval between data collection based on the size of the compound library and the number of concentrations tested as well as the time taken to collect data using both technologies in the same well, while 40 hours is the maximum allowed time frame of exposure due to the potential for evaporation using the 1536-well plate format. The positive control, tetra-N-octylammonium bromide (Sigma, CASRN = 14866-33-2), in titration (16 concentrations, 2.35 nM to 77μM, for plate to plate quality control), was dispensed on each plate.

### Concentration-response data processing

The raw plate reads for each titration point were first normalized relative to the DMSO only wells on each plate (% Activity = [(V_compound_ − V_DMSO_)/ V_DMSO_)] × 100, where V_compound_ denotes the compound well values, and V_DMSO_ denotes the median values of the DMSO-only wells. The % activity (either % of cell death increase or % of cell viability decrease) was rescaled so that the baseline value is 0%. The normalized value was corrected by applying a pattern correction algorithm using DMSO control plates stacked between the screening plates [18]. The normalized concentration-response data for each compound at each time point were applied to a qHTS noise filtering algorithm [19,13] with assay noise level (25%, in 2 cell lines x 2 assay technologies) derived from the response variation in the 88 technical replicates on each plate [20] The weighted area-under-curve (wAUC, total activity) [13], point-of-departure (POD, concentration at which the response was equivalent to the noise threshold), AC_50_ (half maximal activity concentration), and E_max_ (maximal response) were determined for each curve. Curves with wAUC > 0 were considered as having significant responses; curves with wAUC = 0 were considered as having no response. The POD and AC_50_ values from the inactive curves were set as the highest tested concentration. The median of activity value was reported for the technical replicates. Activity values are reported for 9667 chemicals in the four assays at https://dataverse.unc.edu/dataset.xhtml?persistentId=doi:10.15139/S3/12321. For the *flor* assay technology, since the increase of wAUC could be affected by the number of cells in the well, the cell doubling factor (adjusted wAUC = original wAUC/ 2^(time point /cell doubling time)) was applied to correct the wAUC. Under the experimental conditions used, the cell doubling time for the HEK293 and HepG2 cell lines is 22 and 40 hours, respectively.

### Cytotoxicity kinetics analysis

**Fig 2:**
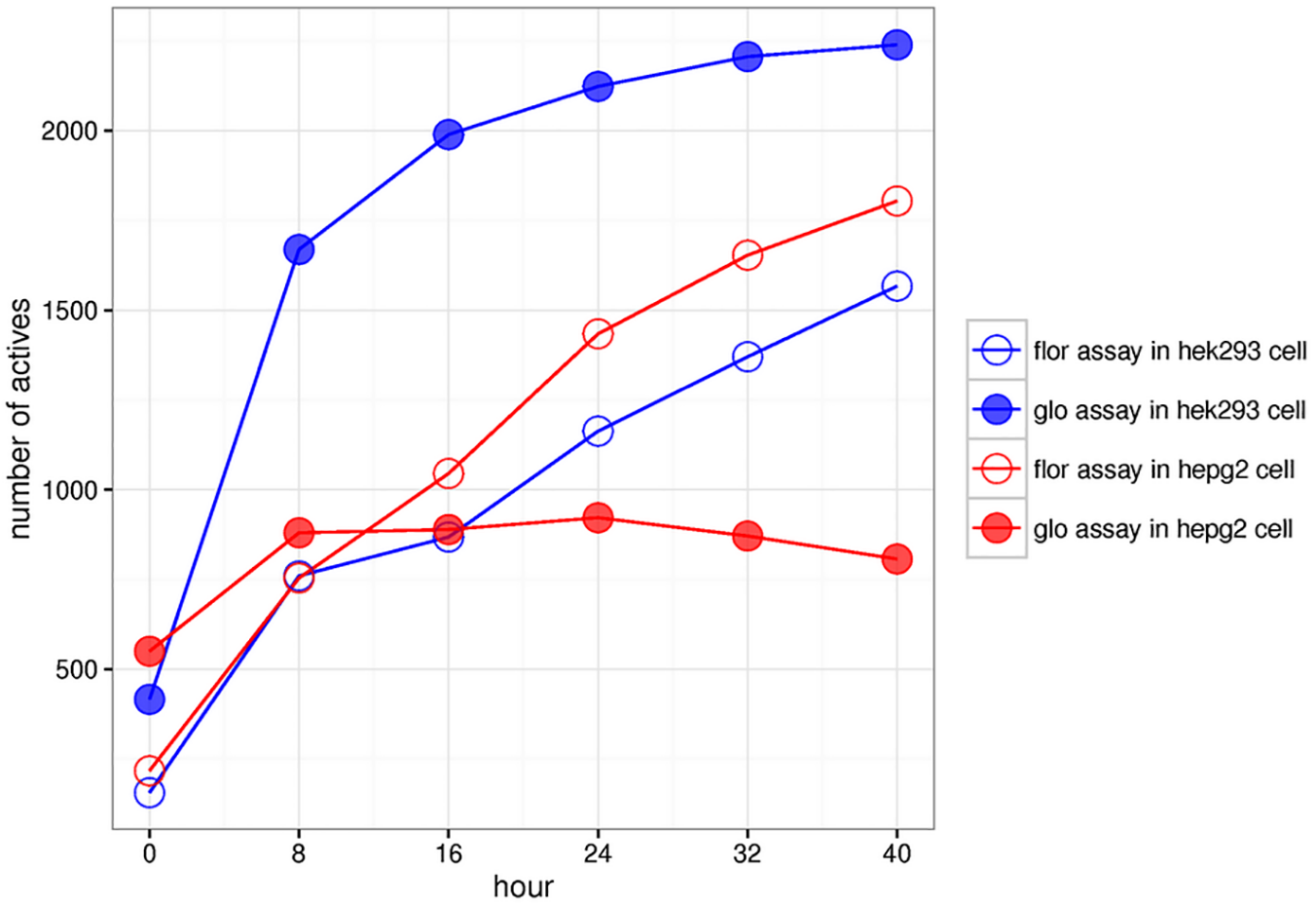
Examples of two chemicals (digitonin *vs*. mitomycin C) with different kinetics of cytotoxicity. Blue: HEK293; red: HepG2. Filled circle (*glo*); hollow circle (*flor*); the arrow represents the earliest time interval where the maximum cytotoxic effect was obtained (filled arrow head: *glo*; hollow arrow head: *flor*).

For each of the four assays (2 cell lines x 2 assay technologies), the Mann-Kendall Trend Test [R package: trend [21]] was applied to identify if there was a monotonic increase in cell death or decrease in cell viability across the different time points for each chemical. The degree of cell death or cell viability was quantified by the wAUC. A p-value < 0.05 was set as the significance threshold. If there was a significant increase in cell death or decrease in cell viability across time points, the earliest time interval at which the maximum cytotoxic effect achieved was determined. The change of effect was quantified by the coefficient of variation (*cv* [22], the ratio of the standard deviation (σ) to the mean μ, σ/μ) based on the wAUC values across time points: 0 to 40 hour (6 wAUC values from 6 experiments), 8 to 40 hour (5 wAUC values), 16 to 40 hour (4 wAUC values), 24 to 40 hour (3 wAUC values), and 32 to 40 hour (2 wAUC values). If the *cv* was smaller than a threshold, the earliest time interval was reported as the time interval where the maximum cytotoxic effect was obtained. The *cv* threshold was set as 0.71 (HEK293[*flor*]), 0.52 (HEK293[*glo*]), 0.76 (HepG2[*flor*]), and 0.85 (HepG2[*glo*]), which was the median *cv* based on the data of the active technical replicates across time points assuming that the replicate data at each time point should have same wAUC. Using digitonin in HEK293[*glo*] as an example (see Fig 2), the *cv* for 0 to 40 hour was 0.51, for 8 to 40 hour is 0.12, for 16 to 40 hour is 0.09, for 24 to 40 hour is 0.006, for 32 to 40 hour is 0.007. Since the *cv* for 0 to 40 hour was 0.51, smaller than the threshold (0.52), it was considered that for this assay (HEK293[*glo*]), digitonin already reached its maximum cytotoxic effect in the time interval of 0 to 8 hour. For chemicals where a monotonic trend was absent (e.g., only having a small differentiation in response between time points or chemicals only active in the later time points), they were grouped depending on their *cv* of 0 to 40 hour or *cv* of 8 to 40 hour and whether there was an effect seen at the 24-, 32-, and 40-hour time points. A flowchart for this process is provided in S1 Fig.

### Enrichment analysis and clustering

The Tox21 chemicals were grouped based on their cytotoxicity kinetics profiles (i.e., the earliest time interval where a maximum cytotoxic effect was obtained in each of the four assays or inactive label). A Fisher’s exact test [23] was conducted on each of the groupings to investigate the association between pairs of individual chemical cytotoxicity grouping and individual Tox21 Phase II assay (https://tripod.nih.gov/tox21/assays/ and [13]). The targets in these assays were either stress response pathways (e.g., TP53 activation) or nuclear receptor related pathways (e.g., estrogen receptor). Activities resulted from various kinds of assay interference such as auto-fluorescence/quenching and chemically-induced cytotoxicity were flagged and not used in the enrichment analysis. Specifically, we flagged activities where the potency (EC_50_) data or total effect data (wAUC) in the Tox21 targeted assays were not significantly greater than the respective data from the Tox21 assays for detecting auto-fluorescent compounds or the cell viability assays multiplexed with the targeted assay [13]. The complete list of assay names is provided in Table A in S1 Text, and include 29 nuclear receptor related assays and 12 stress response pathways. A Bonferroni correction [24] was applied to p-values generated for each chemical cytotoxicity kinetics grouping and the significant threshold was set as 0.05.

The binary association (significant or non-significant) between pairs of individual chemical cytotoxicity kinetics grouping and individual stress response/nuclear receptor pathways were further transformed. The degree of association between the earliest time interval where chemicals reached their maximum cytotoxic effect and activity in these pathway assays was quantified by dividing the sum of times of association of a certain activity at a particular time interval in an assay by sum of times of association of that activity to cytotoxicity kinetics groupings. For example, increased γ-H2AX activity could be associated with ‘x’ different cytotoxicity kinetics groupings and the time interval (0 < time < 8 hours, HepG2[*glo*]) was found ‘y’ of ‘x’ times; thus, the degree of association between 0 < time < 8 hours, HepG2[*glo*] and γ-H2AX induction is ‘y/x’.

For chemical clustering, the wAUC, POD, and E_max_ values per chemical at each time point in each assay were collected. The wAUC value was transformed using log_10_(wAUC+1) function (+1 is to avoid the infinity value for the inactives). The five *cv* values and the log_10_(p-value) from the trend test were also used as descriptors. The values were Z-score normalized. The Pearson’s correlation coefficient value (Pearson’s R) between chemicals was calculated. The chemical-chemical pairwise Pearson’s R values were used in the hierarchical clustering based on the Euclidean distance. The matrix of descriptors used in clustering is provided in the S1 Dataset and on the UNC Dataverse (https://dataverse.unc.edu/dataset.xhtml?persistentId=doi:10.15139/S3/12321).

### Repeated measure linear model

Repeated measure linear model [25] was adopted to explore the source of activity variation based on the activity data from the 24 measurements (2 cell lines x 2 technologies x 6 time points) while accommodating the within-time point correlation. The activity outcome (log_10_(wAUC+1)) was set as a dependent variable; the cell line and technology were included in the model as categorical independent variables; time (with 6 time points) was viewed as a continuous independent variable. The maximum likelihood method with classical assumptions for the random errors (type III sum of squares F statistics) was applied to estimate the degree of activity variation contributed by the three factors: cell line, technology, and time.

## Results

### Screening performance

The two assay technologies performed well in both cell lines. The qHTS assay performance statistics are as follows: signal to background (S/B) ratio from 3 to 16 fold, coefficient of variance (CV) of 6% to 8%, and Z’ factors >0.7 (Table B in S1 Text). Overall, the standard deviation (SD) of half-maximal effect or inhibitory concentration (EC_50_ or IC_50_, respectively) of the positive control tetra-N-octylammonium bromide titration embedded on each plate was <3-fold. In the HEK293 cell line using the *glo* assay, the IC_50_ values of tetra-N-octylammonium bromide were 5.91±1.26 (mean and SD), 2.53±0.74, 1.34±0.52, 0.87±0.44, and 0.79±0.32 μM at 8, 16, 24, 32, and 40 hours, respectively, while using the flor assay, the EC_50_ values of the positive control chemical were 9.88±2.10, 8.01±1.52, 6.06±1.11, 6.80±1.47, and 7.69±1.76 μM at 8, 16, 24, 32, and 40 hours, respectively. Tetra-N-octylammonium bromide had similar potencies in the HepG2 cell line in both assays with IC_50_ or EC_50_ values of 6 to 8 μM (Table B in S1 Text). EC_50_ or IC_50_ data are not available for the 0 time point as the exposure duration was not long enough for the positive control to have an effect. It is interesting to note that among the cell line/technology combinations, the potency of the positive control in the HEK293[*glo*] assay increased with increasing exposure duration while in the other three combinations, potency stayed relatively constant across time.

### Relative assay sensitivity

#### Activity

**Fig 3:**
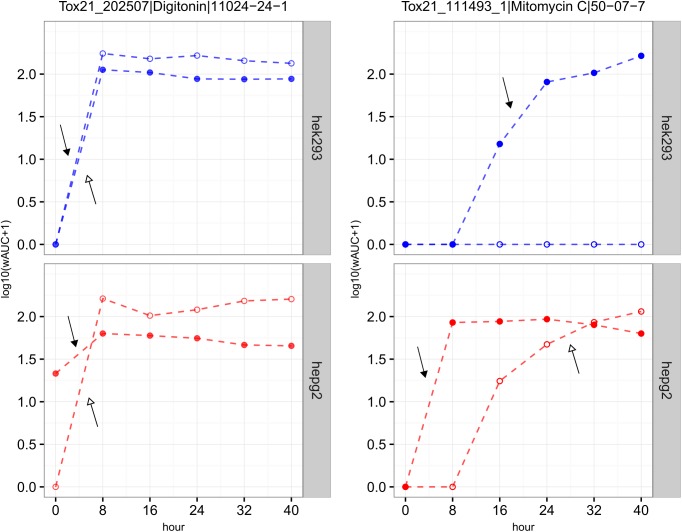
Comparison of number of actives in four assays. The number of actives detected in the four assays at the six different time points. Blue: HEK293 cell line; red: HepG2 cell line. Filled circle: *glo* assay technology; hollow circle: *flor* assay technology.

The number of actives at each time point for each technology and cell line used is presented in Fig 3. Between the four assays, screening chemicals using the HEK293[*glo*] assay resulted in the most number of actives: over 2200 chemicals were considered active at the 40-hour time point. In contrast, using the same assay technology (*glo)* in the HepG2 cell line produced the least number of actives (i.e., only ~800 chemicals were considered active at the 40-hour time point). In fact, across all time points >0 hours, more actives were detected in the HEK293 cell line than in the HepG2 cell line when using the *glo* technology. However, the opposite was observed for the *flor* technology (i.e., more actives were detected in the HepG2 vs. the HEK293 cell line). Moreover, in the HepG2[*glo*] assay, the rate at which the number of actives increased with increasing time was the slowest in comparison with the other three assays. We evaluated the extent of active chemical overlap between the four assays at the 40-hour time point (Table 1). At this maximal exposure time point, 3140 unique chemicals were classified as active in the two cell lines using the two technologies; 1260 (~40%) of which were concordant across the four assays. The HEK293[*glo*] assay produced the most number of unique actives (25%, 791/3140) while the HepG2[*glo*] assay contributed only 1.7% (54/3140) of the unique actives. On the other hand, the percentages of unique actives from the HEK293[*flor*] and HepG2[*flor*] assays were similar (<10%, 273/2140 *vs*. 322/2140, respectively). We also compared overlapping actives between assay technologies (or cell lines) in different cell lines (or assay technologies) (S2 Fig). Over 90% of actives in HepG2[*glo*] assay was also active in HEK293[*glo*] assay.

**Table 1:** Number of active chemical overlaps between four assays at 40 hours classified by cell lines and technologies.

		cell line		
		HEK293	Both	HepG2
**technology**	glo	791	53	54
	Both	153	1260	20
	flor	273	214	322

Pearson’s correlation coefficient (Pearson’s R) was applied to evaluate the activity (POD) correlation between the four assays (Table 2). The results from the *flor* assay technology using either HEK293 or HepG2 cell lines are the most correlated (R = 0.86) while the results of the HEK293[*glo*] technology is less correlated compared with the other three assays, with R value ranging from 0.6 to 0.7. In addition, we applied the repeated measure linear model to the wAUC data to estimate the degree of activity variation contributed by the three factors: cell line, technology, and time. The result shows that technology contributes the major variation of activity (65.6%) among the explainable variation, while time contributes 20% and the cell line contributes around 14.4%.

**Table 2:** Activity correlation between four assays.

	hek293_flor	hek293_glo	hepg2_flor	hepg2_glo
hek293_flor	1.00	0.60	0.86	0.76
hek293_glo	0.60	1.00	0.64	0.70
hepg2_flor	0.86	0.64	1.00	0.78
hepg2_glo	0.76	0.70	0.78	1.00

To evaluate at which time point the total number of actives became stabilized, the fold change of the number of actives between two consecutive time points was calculated (Table C in S1 Text). A fold change threshold for each assay technology was derived based on the number of actives at the 0-hour time point, assuming that only the physicochemical properties of the chemicals and the technical assay variation contributed to the observed effect. For example, for the *glo* technology, the number of actives at 0-hour time point in HEK293 cell line and in HepG2 cell line were 416 and 550, respectively. Comparing to the average number (483), there was < 20% of increase (or decrease) of number of actives using either of the cell lines (e.g., 550/483 = 1.14 [HEK293] vs. 416/483 = 0.86 [HepG2]). Thus, 20% is set as the threshold to define if there is a significantly increase of actives when comparing two consecutive time points (Table C in S1 Text). Based on the threshold, we identified the time point where the total number of actives became stabilized is 16 and 32 hours in the *glo* and *flor* assays, respectively.

#### Kinetics of cytotoxicity

In each of the four assays, the chemicals were grouped as shown in S1 Fig. The log_10_(wAUC+1) of two chemicals across the six time points are provided as examples in Fig 2. Digitonin, a mild detergent, quickly reaches its maximum cytotoxic effect (0 < time < 8 hour) in all four assays. On the other hand, mitomycin C (MMC), a genotoxic compound that induces DNA cross-links, produces an assay specific cytotoxic effect (i.e., it was not active in the HEK293[*flor*] assay, but was in the HepG2[*flor*] assay with a relatively slow rate to reach its maximum cytotoxic effect (i.e., 24 < time < 32 hour). Although MMC was active in both the HEK293[*glo*] and the HepG2[*glo*] assays, it reached its maximum cytotoxic effect more quickly in the HepG2 cell line (0 < time < 8 hour) compared to the HEK293 cell line (16 < time < 24 hour). The number of chemicals that reach their maximum cytotoxic effect in each assay time interval is provided in Table 3. Greatest sensitivity to time was seen in the HEK293[*glo*] assay (i.e., the number of active chemicals reaching their maximum cytotoxic effect was more scattered over the different time intervals). For the same assay technology, over 75% of actives in the HepG2[*glo*] assay quickly reached their maximum cytotoxic effect (0 < time < 8 hour). Overall, in the *flor* technology, there were more chemicals that required longer times to reach their maximum effect (~ 20%, time >32 hour) than by using the *glo* technology (< 10%, time >32 hour). And likewise, the results using the *flor* technology were more similar: ~40% of actives quickly reached their maximum effect (0 < time < 8 hour) in both cell lines. In total, 1960 chemicals showed varying cytotoxicity kinetics in the four assays (including inactive labels) while 5960 chemicals were non-cytotoxic in all four assays under the experimental conditions used.

**Table 3:** Number of chemicals that reached their maximum cytotoxic effect in each time interval by assay.

assay	0 < time < 8	8 < time < 16	16 < time < 24	24 < time < 32	32 < time < 40	time > 40^*^	inactive	inconclusive
flor[HEK293]	523 (42.9%)	103 (8.4%)	203 (16.6%)	127 (10.4%)	223 (18.3%)	41 (3.4%)	7927	520
glo[HEK293]	545 (25.9%)	664 (31.5%)	424 (20.1%)	248 (11.8%)	151 (7.2%)	73 (3.5%)	6999	563
flor[HepG2]	609 (40.2%)	178 (11.8%)	330 (21.8%)	137 (9.0%)	241 (15.9%)	19 (1.3%)	7774	379
glo[HepG2]	586 (74.9%)	71 (9.1%)	57 (7.3%)	23 (2.9%)	31 (4.0%)	14 (1.8%)	8206	679

(percentage) represents the percentage of chemicals identified in this time interval relative to the total number of chemicals identified in all time intervals

*: the time interval that cytotoxic effect become stabilized is outside the exposure time

### Identification of cytotoxicity dependent toxicity pathway linkage

**Fig 4:**
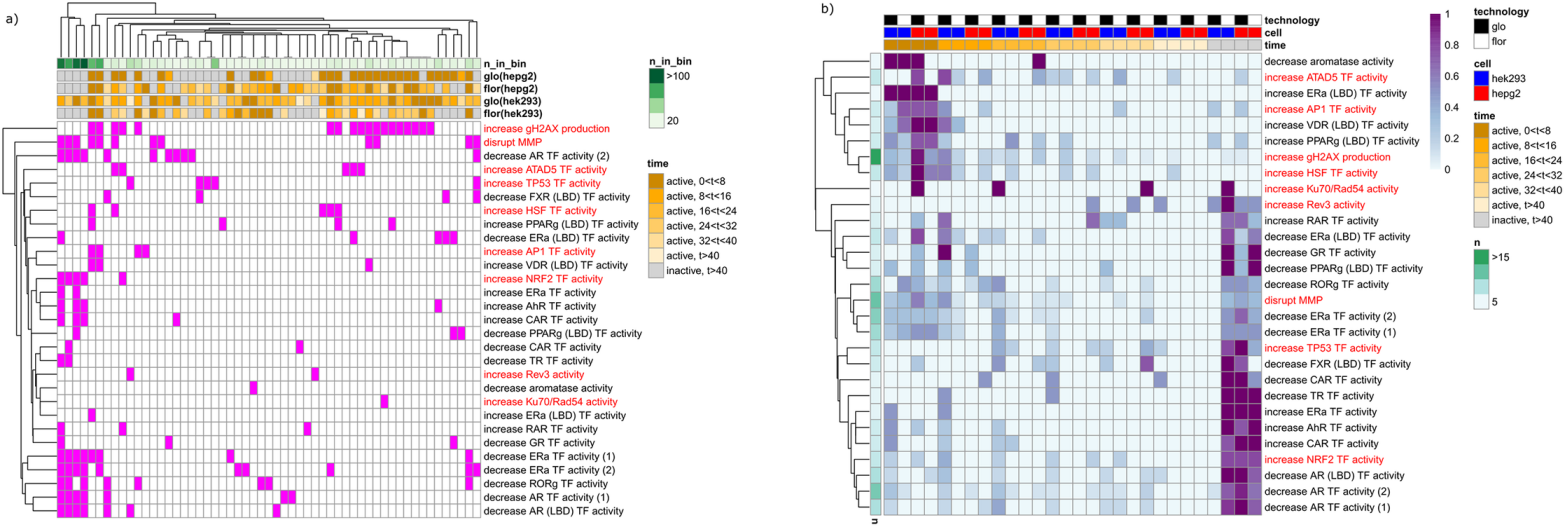
Toxicity pathway clustering based on chemical cytotoxicity kinetics data. a) binary association between activities in pathways (rows) and chemical cytotoxicity kinetics groupings(columns). Magenta cell: significant; white cell: non-significant. n_in_bin: number of chemicals in the bin (grouping). b) degree of association between the earliest time intervals where chemicals reached the maximum cytotoxic effect and activity in toxicity pathways. Red row text: stress response pathways; black row text: nuclear receptor related pathways; n: number of times of associations found in a); more intensified purple color, higher degree of association.

The 1960 chemicals can be grouped into 350 bins based on their cytotoxicity kinetics profiles in the four assays (e.g., digitonin belongs to the bin that is labelled with “0 < time < 8” in all four assays and there are 73 chemicals in this group). The enrichment analysis was conducted as described in the Methods section using the results from the primary screen of each Tox21 assay [13]. The binary significance of association for each bin-toxicity pathway pair are presented as a heat map using hierarchically clustering with average linkage (Fig 4A). Nine of 29 nuclear receptor related pathways and three of 12 stress response pathways did not have any significant associations with any of the cytotoxicity kinetics groupings, which includes most of agonist-mode of nuclear receptor related assays and assays related to activation of HIF-1, NF-κB, and ATF-6.

Chemicals that increased production of γ-H2AX were found to be most commonly associated with the cytotoxicity kinetics grouping, and were particularly associated with the groups where chemicals show significant activities in all four assays. Chemicals that caused mitochondrial membrane potential (MMP) disruption were found to be the second group most commonly associated with the cytotoxicity kinetics grouping, specifically with two groups of chemicals: chemicals that induced cytotoxic effects in all four assays or chemicals that active in the HEK293[*glo*] assay only. In contrast, chemicals that were active in nuclear receptor related pathway assays were preferentially associated with chemical groups that induced cytotoxic effects in the HEK293[*glo*] assay only. In addition to the aforementioned two stress response pathways (MMP and γ-H2AX), other cytotoxicity kinetics groupings were found to be to be associated with chemicals that activated other stress response pathways. For example, chemicals that increased TP53 transcriptional factor (TF) activity were associated with groups where chemicals required a longer time to reach the maximum cytotoxic effect (time > 16 hours).

The degree of association between the time interval where chemicals reached their maximum cytotoxic effect and activity in the stress response/nuclear receptor pathway assays was calculated (see Methods). For example, chemicals that increased γ-H2AX activity were associated with 18 different cytotoxicity kinetics groupings and the time interval (0 < time < 8 hours, HepG2[*glo*]) was found 17 of 18 times; thus, the degree of association between 0 < time < 8 hours, HepG2[*glo*] and γ-H2AX induction is 0.94 (17/18)). The degree of association is presented as a heat map using hierarchical clustering (only the rows) with average linkage (Fig 4B). Several Tox21 stress response/nuclear receptor pathways were found to be clustered together through the association of active chemicals to certain cytotoxicity kinetics groupings. For example, the chemicals that decreased androgen receptor (AR) TF activity in either the full-length receptor or the ligand-binding-domain (LBD) assays tended to be more selectively active in the HEK293[*glo*] assay, similar to the chemicals that increase Nrf2 TF activity. Other linkages were revealed (the list was based on the decreasing number of times of association found; the number of times is provided in parenthesis): for example, increased production of γ-H2AX (18) clustered with increased heat shock factor (HSF) TF activity (5), decreased estrogen receptor (ER) TF activity (10) clustered with disruption of MMP (12), increased TP53 TF activity (5) clustered with decreased farnesoid X receptor (FXR) TF activity (4), increased aryl hydrocarbon receptor (AhR) TF activity (4) clustered with increased constitutive androstane receptor (CAR) TF activity (4), decreased peroxisome proliferator-activated receptor (PPARγ) TF activity (4) clustered with decreased glucocorticoid receptor (GR) TF activity (3), increased Activator protein 1 (AP-1) TF activity (4) clustered with increased vitamin D receptor (VDR) TF activity (3). Some of the associations could also be seen by comparing the overall activity similarity of chemicals between the Tox21 assays (e.g., increased AhR TF activity clustered with increase CAR TF activity, increased production of γ-H2AX clustered with increased HSF TF activity, and decreased ER TF activity clustered with disruption of MMP) but some are not (S3 Fig), indicating the associations seen in Fig 4B are more related to the underlying mechanisms of cytotoxicity. In addition, some Tox21 stress response/nuclear receptor pathways were not directly linked with any other pathway such as the increase of ATAD5 TF activity.

### Linkage investigation and chemical prioritization

**Fig 5:**
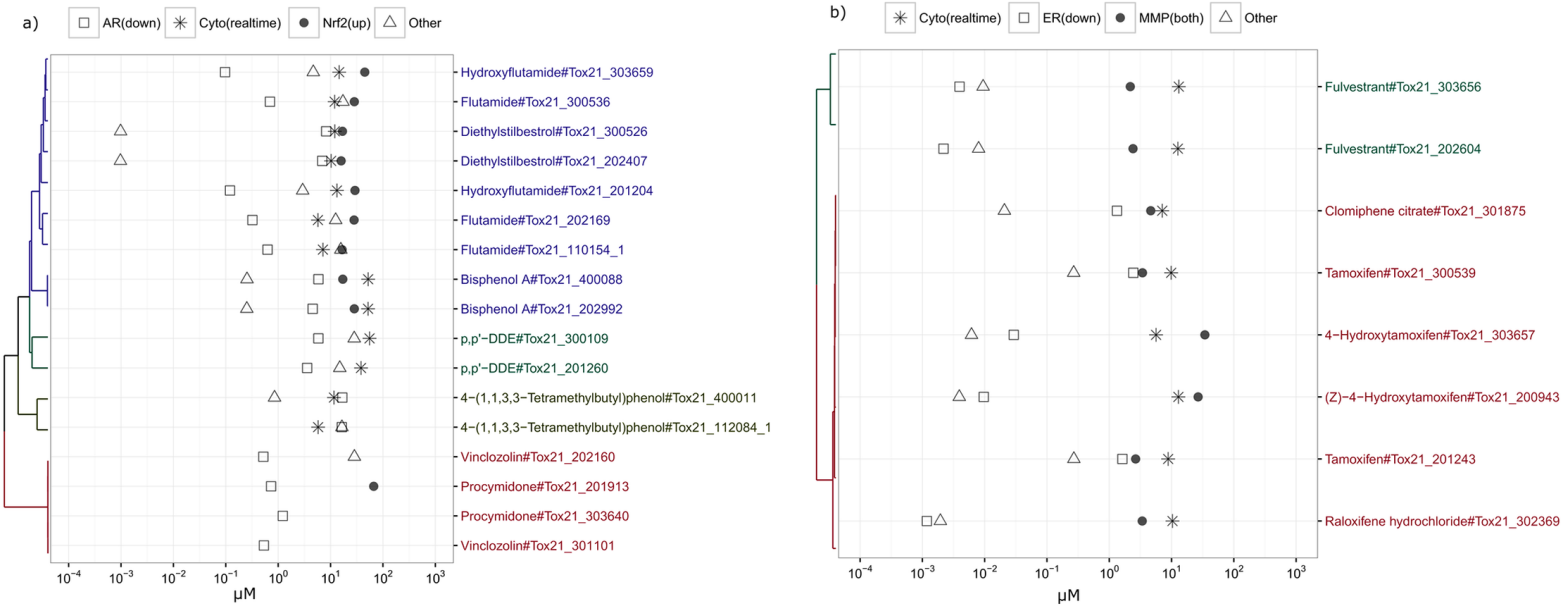
Activity potency comparison between the known nuclear receptor antagonists clustered based on their cytotoxicity kinetics activity data. a) AR antagonists. b) ER antagonists. Dendrogram on the left represents the clustering using the cytotoxicity kinetics activity data and color represents the groupings; the symbols represent the most potent activity in the respective assays.

As noted above, chemicals that decreased AR TF activity tended to be more selectively active in the HEK293[*glo*] assay, similar to the chemicals that increased Nrf2 TF activity. To investigate this association, the cytotoxicity kinetics activity data of AR antagonists with well documented effects in humans or rodents in the Tox21 10K library (including both pharmaceuticals and environmental chemicals) [26] were hierarchically clustered with average linkage (k = 4, number of groups) and the POD potency data related to decreased AR TF activity (AR(down)), increased Nrf2 TF activity (Nrf2(up)), other activities in Tox21 (Other), and cytotoxicity in the four assays (Cyto(realtime)) in this study compared (Fig 5A and S4A Fig). When there were multiple activities in the real-time cytotoxicity data, the most potent activity is presented. The four AR antagonists (hydroxyflutamide, flutamide, diethylstilbestrol, and bisphenol A) with increased Nrf2 TF activity were grouped together due to their similar cytotoxicity kinetics activity data (i.e., active only in HEK293[*glo*]). Weak Nrf2 activity was seen for procymidone; however, this effect was inconsistent across different chemical sources (e.g., chemicals from different vendors/preparations) for this compound. The POD potency of Nrf2 activation was within 10-fold of the POD potency of cytotoxicity (in this case, HEK293[*glo*]) in the real-time assays, while the AR activity tended to be more potent than both the activation of Nrf2 and the cytotoxic effect in HEK293[*glo*] (purple cluster including hydroxyflutamide in Fig 5A). The relationship between the cytotoxicity kinetics data, decreased AR TF activity, and increased Nrf2 TF activity matches with the profile in Fig 4B. The two AR antagonists that had no activity in all four real-time cytotoxicity assays (vinclozolin and procymidone), showed very few and weak activities in the Tox21 assays other than their AR-related activities. The others (p,p'-DDE and 4-(1,1,3,3-tetramethylbutyl)phenol)) tended to quickly reach the maximum cytotoxic effect in at least three assays and had more diverse activities seen in other Tox21 assays. The same exercise was also performed on the known ER antagonists in the Tox21 10K library (k = 2, number of groups)[27](Fig 5B and S4B Fig). Fulvestrant had distinct cytotoxicity kinetics when compared with the other ER antagonists, and all of them showed activity in the Tox21 MMP assay (either increase or decrease of MMP). The potency of MMP disruption tended to track with cytotoxicity potency (for ‘tamoxifen’ cluster’, potency was from all four active assays; while for the fulvestrant, the potency was from the solely active, HEK293[*glo*] assay). The more diverse cytotoxicity kinetics profiles in relationship with disruption of MMP and decrease of ER TF activity were also reflected in the results seen in Fig 4. The above analyses suggest that there are classes of AR/ER antagonists having Nrf2 activation/MMP disruption at a potency level similar to the potency of their cytotoxicity effects in the HEK293/HepG2 cell lines.

Thus, considering the relationship as a biological feature that can help to identify true AR/ER antagonists, we prioritized the actives identified in previous Tox21 screens. In total, there are 108 chemicals with decreased AR TF activity and increased Nrf2 activity and 70 chemicals with decreased ER TF activity and MMP disruption having cytotoxicity kinetics profile as seen in the Fig 4B based on the hierarchical clustering analysis. The results were ranked by their most potent AR/ER activity and the respective Nrf2/MMP activity as well as the activity in the real-time cytotoxicity assays are reported in S5 Fig and S1 and S2 Tables.

## Discussion

In this study, we interrogated chemical-induced cytotoxicity at six different time points (0, 8, 16, 24, 32, & 40 hours) using two cell lines (HEK293 and HepG2) and two real-time assay technologies measuring different cytotoxicity mechanisms: the *glo* technology measured the reducing potential of cells and thus their metabolic ability (i.e., decrease of cell viability) while the *flor* technology measured the loss of cellular membrane integrity (i.e. increase of cell death). Based on the number of actives detected and the kinetics of cytotoxicity observed, greatest sensitivity was seen in the HEK293[*glo*] assay as this assay has the most number of actives as well as the most number of unique actives. It also has the greatest temporal variation. On average, actives in the HEK293[*glo*] assay tended to be ~2 fold more potent than the same chemicals when active in the HepG2[*glo*] assay, which might explain why more actives were detected using the HEK293[*glo*] assay. In contrast, the potency of actives in the two cell lines using the *flor* assay technology was more similar. Using the Promega CellTiter-Glo® assay, which assessed cytotoxicity by measuring ATP levels, Xia *et al*. [8] also showed that HEK293 cells were more sensitive than HepG2 cells in terms of a greater number of active compounds and greater potency. The greater sensitivity in HEK293 (vs HepG2) might be related to the faster doubling time of the HEK293 cell line (22 hours vs. 40 hours for the HepG2 cell line) while the greater sensitivity in *glo* technology (vs *flor)* is related to the use of a metabolic activity marker as the endpoint vs a compromised cell membrane as the endpoint. Metabolic activity markers can detect non-lethal perturbations (e.g., cessation of proliferation and inhibition of mitochondrial respiration) in contrast to the *flor* technology that detects more “*bona fide*” cell death (a lack of cell membrane integrity) [5].

By analyzing the degree of variation contributed by three factors (technology, time, and cell line) by repeated measure linear model, we found the technology factor contributed the most (>60%) to the variation in activity while the other two factors (time and cell line) each contributed <20%. However, interactions were seen between these three factors, especially the cell line by technology interaction effect, which means that each assay should be treated independently. Future study design could take the interaction effect into account with application of a more complex statistical model for a better assessment of the source of variation. Nevertheless, the current analysis demonstrated that the difference of these two technologies affected the results the most.

In terms of kinetics, we found that a longer exposure time was needed in order to obtain the most number of actives using the *flor* technology (32 hours vs 16 hours using the *glo* technology) and more actives required a longer exposure time to reach the maximum cytotoxic effect by the *flor* technology (~ 20%, time >32 hour vs < 10%, time >32 hour by the *glo* technology). This observation is most likely related to the different cytotoxicity mechanisms detected by these two assay technologies: the *flor* technology detects cell membrane leakage, which is the last step of the necrosis while the *glo* technology detects cell metabolic perturbations, which happen after the chemical insult and would occur before cell membrane leakage.

Based on the assumption that chemicals with similar cytotoxicity profiles can have similar MOA, we grouped the chemicals based on their similarity of cytotoxicity kinetics profiles. To take into account the correlated structure of the time factor, we designed a parameter that captured the earliest time interval where a maximal cytotoxic effect was achieved, assuming that the cytotoxic effect had a monotonic behavior during the exposure duration (40 hours, the maximum allowed exposure duration using the 1536-well plate format due to the potential for evaporation). We also assumed that the MOAs might be represented by some of the activities in Tox21 stress response/nuclear receptor assays. Fisher's exact test was conducted in order to identify the significant associations between pairs of individual cytotoxicity kinetics grouping and individual assay. We found that chemicals that increased production of γ-H2AX and/or disrupted MMP were often associated with groups of chemicals that induced cytotoxicity. The results were not surprising since the formation of γ-H2AX plays a key role in responding to DNA double-strand breaks [28] and alterations in MMP is frequently a decisive event for cell death, irrespective of an underlying mechanism that might be apoptotic, necrotic, or autophagic [29]. Chemicals that increased production of γ-H2AX were found to be particularly associated with the groups that quickly reached the maximum cytotoxic effect in all four assays. On the other hand, chemicals that disrupted MMP were not only associated with groups of chemicals that were active in all four assays but also with groups of chemicals that were active in the HEK293[*glo*] assay only, suggesting that the two chemical groups may involve different mechanisms of cytotoxicity [30].

DNA damage is a likely MOA for cytotoxicity. With the exception of increased production of γ-H2AX, the Tox21 genotoxicity related pathway assays, including increase of TP53 TF activity, increase of ATAD5 protein production, increase of Ku70/Rad54 protein activity, and increase of Rev3 enzyme activity, tended to be associated with the groups of chemicals which required a longer time to reach a maximum cytotoxic effect. It has been reported that the formation of γ-H2AX tends to be quite fast, usually less than 4 hour after the start of exposure [31]. On the other hand, chemicals that activated other genotoxicity related pathways involving cell cycle processes (cell doubling time is 22 hours and 40 hours for HEK293 cell line and HepG2 cell line, respectively) would probably take a longer time to reach maximal adverse biological consequences, including cytotoxicity. Only three of 12 available stress response pathways did not have any significant associations with any of the cytotoxicity kinetics groupings; the three assays were those related to activation of HIF-1, NF-κB, and ATF-6. These three assays had the least number of actives compared with the other stress response pathway assays. For nuclear receptor related assays, interestingly, the chemical groups that were only active in the HEK293[*glo*] assay were often associated with chemicals that either increase or decrease nuclear receptor related TF activity.

We further clustered the Tox21 stress response/nuclear receptor pathway assay results based on their degree of association to the different cytotoxicity kinetics labels (i.e., earliest time interval maximal cytotoxic effect achieved), we found that active chemicals in some stress response/nuclear receptor pathways tend to have rather unique kinetics of cytotoxicity (e.g., increase of ATAD5 protein production). However, there were also clusters of pathways where active chemicals exhibited similar kinetics of cytotoxicity. One possible explanation is that the active chemicals in these pathways may share certain chemical structures (i.e., toxicophores) related to the observed cytotoxic effect. For example, the bisphenol structural class that increases Nrf2 activation and decreases AR TF activity. The other possible explanation is that there may be shared underlying biological processes between these pathways which converge on a similar cytotoxic profile. An example of such a biological interaction is that a positive heat shock response could induce phosphorylation of histone H2AX in mammalian cells [32]. In addition, in relation to the HSF and γ-H2AX association, a convergent mechanism of toxicity has been suggested based on the interaction of the DNA damage and heat shock pathways through the DNA Damage checkpoint control protein, MDC1 [33].

When considering the found associations between nuclear receptor pathways and cytotoxicity, it is important to note that there is no endogenous androgen and estrogen receptors in the HEK293/HepG2 cell lines. Hence, the linkages between stress response pathways and nuclear receptor pathways (i.e., Nrf2 activation–decrease of AR TF activity and MMP disruption–decrease of ER TF activity) through cytotoxicity are most likely due to shared toxicophores rather than to a biological interaction between the pathways. For example, the bisphenol structural class that increases Nrf2 activation and decreases AR TF activity. In these cases, although the activity on nuclear receptors cannot be the MOA for cytotoxicity, considering that the nuclear receptors can be viewed as biosensors that mediate the cellular reactions to chemical insult, grouping chemicals based on their activity on the nuclear receptors can still provide useful information and activity on the stress response pathway may serve as the possible MOA for cytotoxicity if both present similar potency values. For *in vitro* HTS assays, it is recognized that each assay only represents a part of the components in the stress response/nuclear receptor pathways [34]. Orthogonal assays are needed to help discriminate the true pathway-level actives from the artifacts. Thus, we think if some of the pathway-level actives also presented the identified relationship (i.e., cytotoxicity dependent toxicity pathway linkages), the relationship could be used as *in vitro* biological features to prioritize chemicals in previous *in vitro* nuclear receptor screenings for further *in vivo* endocrine screening. Preferably, the potency of nuclear receptor activity should be more potent than the potency seen in the real-time cytotoxicity assays. However, since the different cell lines and time points were used in various Tox21 assays, potency in the different assays might not be directly comparable.

Based on this concept, the linkages of decrease AR TF activity–increase Nrf2 TF activity and decrease ER TF activity–MMP disruption were investigated using known nuclear receptor antagonists. The expected relationships were confirmed and actives in the Tox21 assays were prioritized. In addition to the antagonists used in the study, other top actives (based on potency) also have abundant published studies related to AR (e.g., cyproterone acetate, nilutamide, bicalutamide). For chemicals that decreased ER TF activity (S2 Table), the results were compared with the corresponding ER antagonist AUC score if available from the pathway-level model constructed using all Tox21/ToxCast ER assays of 1812 ToxCast chemicals [14]. In addition to the known ER antagonists, some other classes of chemicals overlapped with the chemicals with ER antagonist AUC score higher than 0.01. These include, for example, two gallates with long alkyl chains (octyl gallate and dodecyl gallate), two ionic liquids (hexadecyltrimethylammonium bromide and N,N,N-trimethyltetradecan-1-aminium chloride), and the organotin tetrabutyltin. Other ionic liquids and organotins also appeared in the prioritized ER list (S2 Table). Although mostly caused by the results from the HEK293[*glo*] assay, greater overlap in potency was seen between the ER antagonist activity and the most potent activity in the four real-time cytotoxicity assays, suggesting that there was a greater chance that ER activity could be confounded by cytotoxicity.

In summary, the real-time cytotoxicity screen provides informative cytotoxicity kinetics data to help classify chemicals related to their cytotoxicity MOA, which could be hypothesized by linking the cytotoxicity outcomes with Tox21 cell-based, target-specific data. In the future, with the advance of phenotypic screening using *in vivo* alternative animal models [35,36] and with the better curation of historical animal data [37] with target-level effects, similar studies can be conducted to relate chemical-induced *in vivo* outcome to the respective *in vitro* mechanisms through Tox21 assays or all PubChem assays [38,39]. When using these in *in vitro* assays for MOA identification, limitations such as the difficulty in cross-species/cross-cell line comparison, the over-simplification schema for *in vivo*/*in vitro* dose extrapolation, and limited metabolic activity *in vitro* must be considered.

## Supporting information

S1 Fig Flowchart to identify the earliest time interval where the maximum cytotoxic effect of chemical is reached. (PNG)

S2 Fig The Venn diagram [40] of the number of actives at the 40-hour time point. a) comparison between cell lines using either *glo* or *flor* technology. b) comparison between assay technologies using either HEK293 or HepG2 cell line.
(PDF)

S3 Fig Hierarchical clustering of toxicity pathways based on the activity similarity of chemicals. The log_10_(point-of-departure (POD)) activity value was used when comparing pathways. The 1 molar concentration activity value was set for the inactive and inconclusive chemicals (artifacts included). Pearson’s correlation between toxicity pathways was calculated. Only chemicals active in at least one of the pathways were included. The average linkage was used to connect the pathways with similar activity profile.
(PDF)

S4 Fig Cytotoxicity kinetics of the representative nuclear receptor antagonists in the clusters. **a) AR.** The background color corresponds to the clusters presented in Fig 5A; filled circle (*glo*); hollow circle (*flor*). b) ER. The background color corresponds to the clusters presented in Fig 5B; filled circle (*glo*); hollow circle (*flor*).
(TIF)

S5 Fig Prioritized chemicals based on identified cytotoxicity dependent toxicity pathway linkages. **a)** chemicals that decrease AR TF activities in the any of the three Tox21 assays (tox21-ar-bla-antagonist-p1, tox21-ar-mda-kb2-luc-antagonist-p1, tox21-ar-mda-kb2-luc-antagonist-p2, see Table A in S1 Text for the description). b) chemicals that decrease ER TF activities in any of the two Tox21 assays (tox21-er-luc-bg1-4e2-antagonist-p1, tox21-er-luc-bg1-4e2-antagonist-p2). Median activity in real-time cytotoxicity assays was used. c) chemicals that decrease ER TF activities in any of the two Tox21 assays (tox21-er-luc-bg1-4e2-antagonist-p1, tox21-er-luc-bg1-4e2-antagonist-p2). Most potent activity in real-time cytotoxicity assays was used.
(PDF)

S1 Text **Table A. Tox21 assays used in the enrichment analysis. Public data can be downloaded from** https://tripod.nih.gov/tox21/assays/. **Table B. Assay performance evaluation. Table C. Number of actives at each time point and the fold change of actives between two consecutive time points.**
(DOCX)

S1 Table Activity information of the prioritized potent AR antagonists. (XLSX)

S2 Table Activity information of the prioritized potent ER antagonists. (XLSX)

S1 Dataset Activity values of the Tox21 chemicals. (XLSX)
