# Correction: Real-time cell toxicity profiling of Tox21 10K compounds reveals cytotoxicity dependent toxicity pathway linkage

**DOI:** 10.1371/journal.pone.0181291

**Published:** 2017-07-07

**Authors:** Jui-Hua Hsieh, Ruili Huang, Ja-An Lin, Alexander Sedykh, Jinghua Zhao, Raymond R. Tice, Richard S. Paules, Menghang Xia, Scott S. Auerbach

The images for Figs 2 and 3 are incorrectly switched. The image that appears as Fig 2 should be Fig 3 and the image that appears as Fig 3 should be Fig 2. The figure captions appear in the correct order.

**Fig 2:**
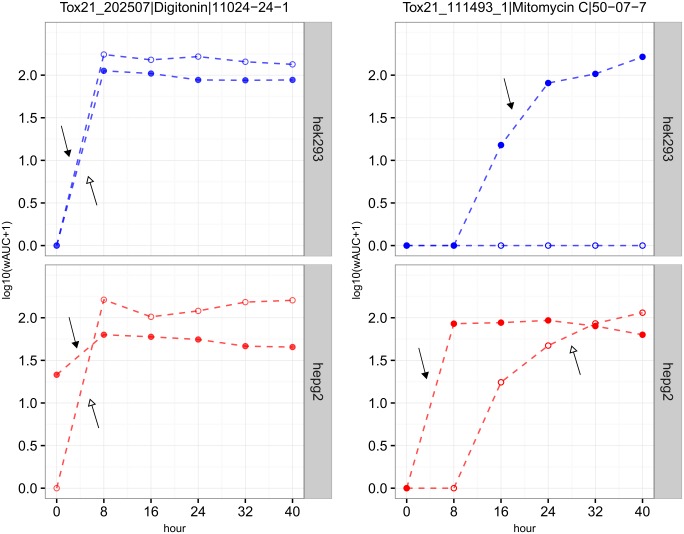
Examples of two chemicals (digitonin *vs*. mitomycin C) with different kinetics of cytotoxicity. Blue: HEK293; red: HepG2. Filled circle (*glo*); hollow circle (*flor*); the arrow represents the earliest time interval where the maximum cytotoxic effect was obtained (filled arrow head: *glo*; hollow arrow head: *flor*).

**Fig 3:**
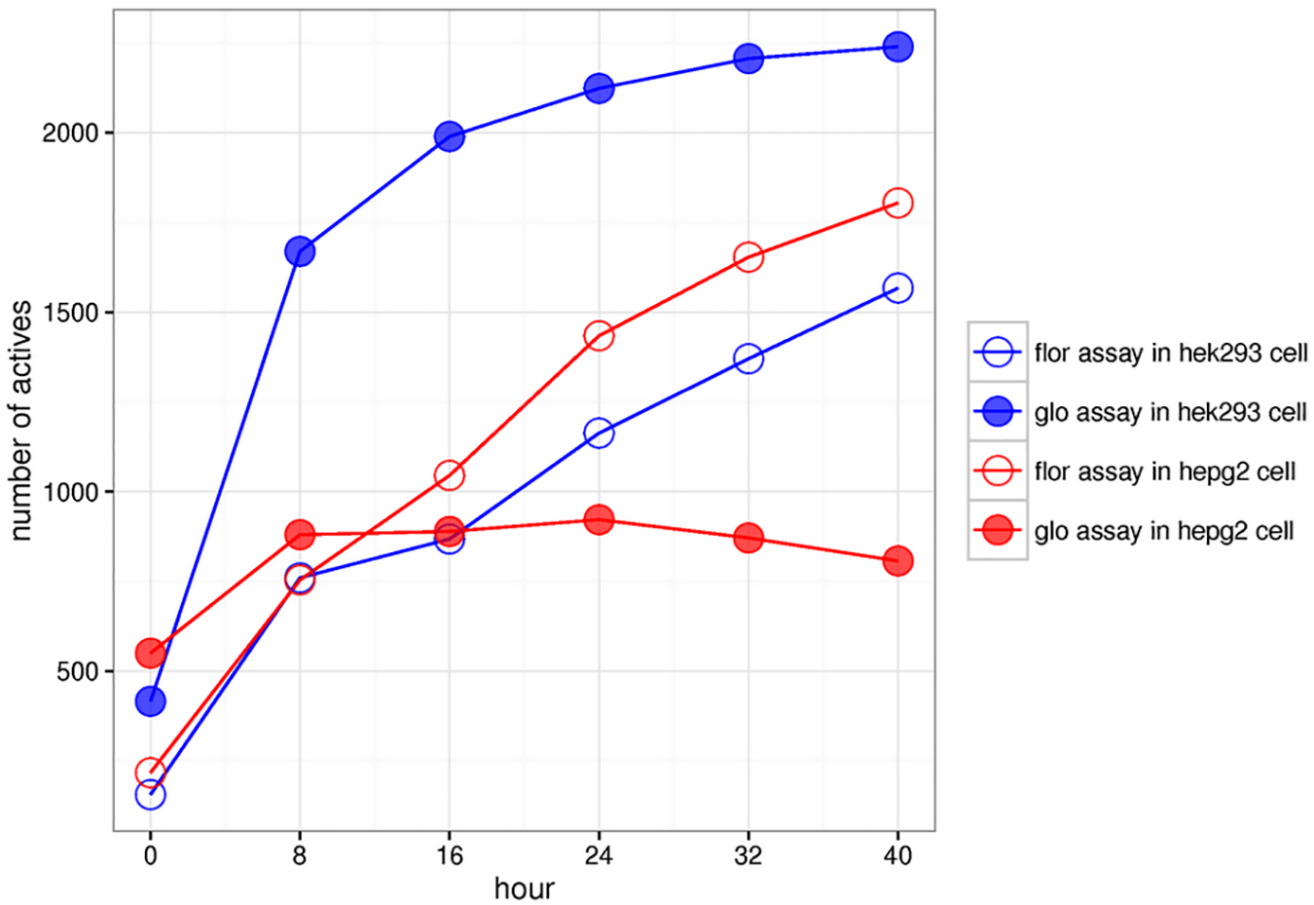
Comparison of number of actives in four assays. The number of actives detected in the four assays at the six different time points. Blue: HEK293 cell line; red: HepG2 cell line. Filled circle: *glo* assay technology; hollow circle: *flor* assay technology.
