# Supplementary figures and images for: Real-time cell toxicity profiling of Tox21 10K compounds reveals cytotoxicity dependent toxicity pathway linkage
Source: PLoS One. 2017 May 22;12(5):e0177902. doi: 10.1371/journal.pone.0177902 (PMC5439695; doi:10.1371/journal.pone.0177902)

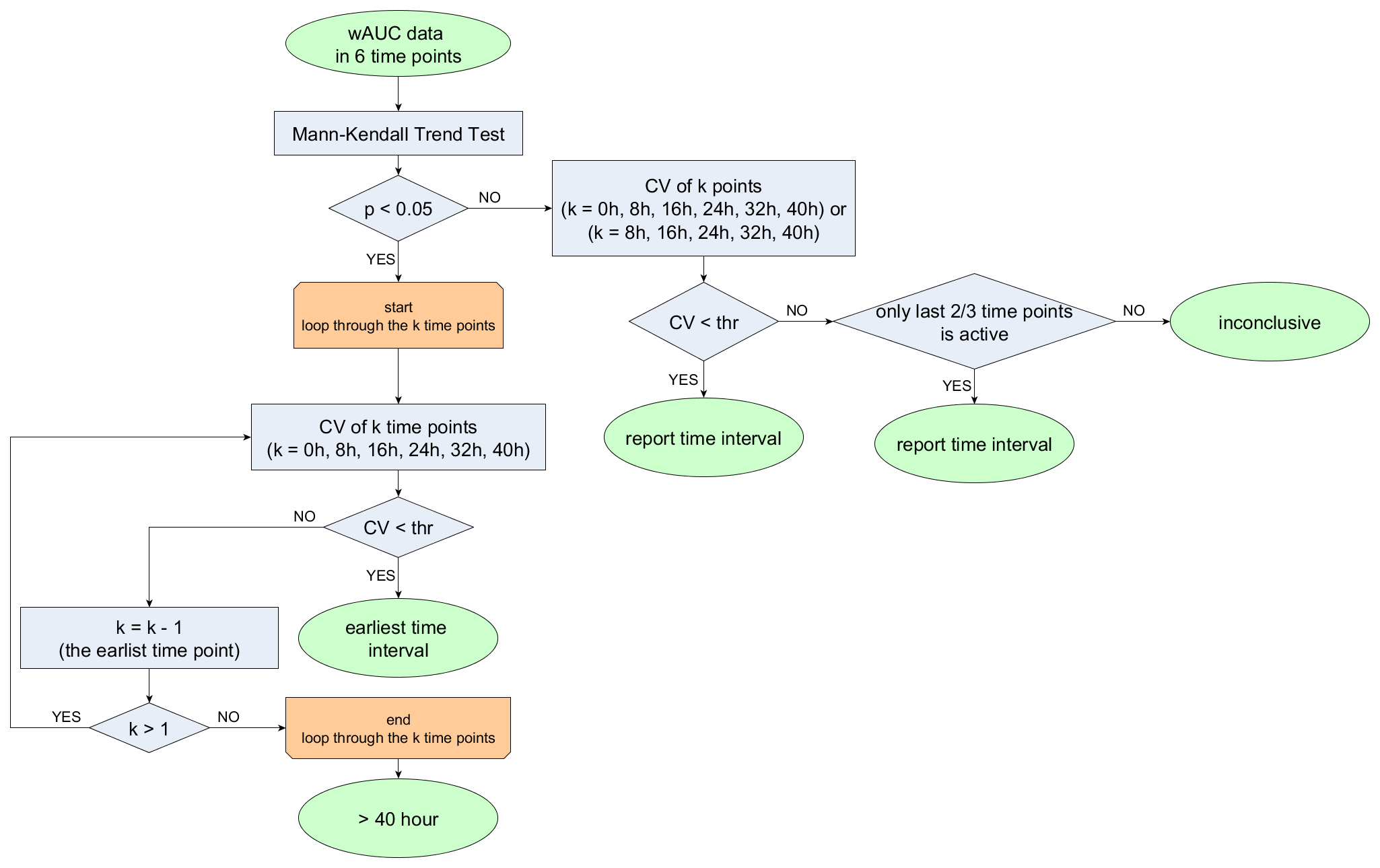

Supplement: S1 Fig — (PNG) [file pone.0177902.s001.png]

a)

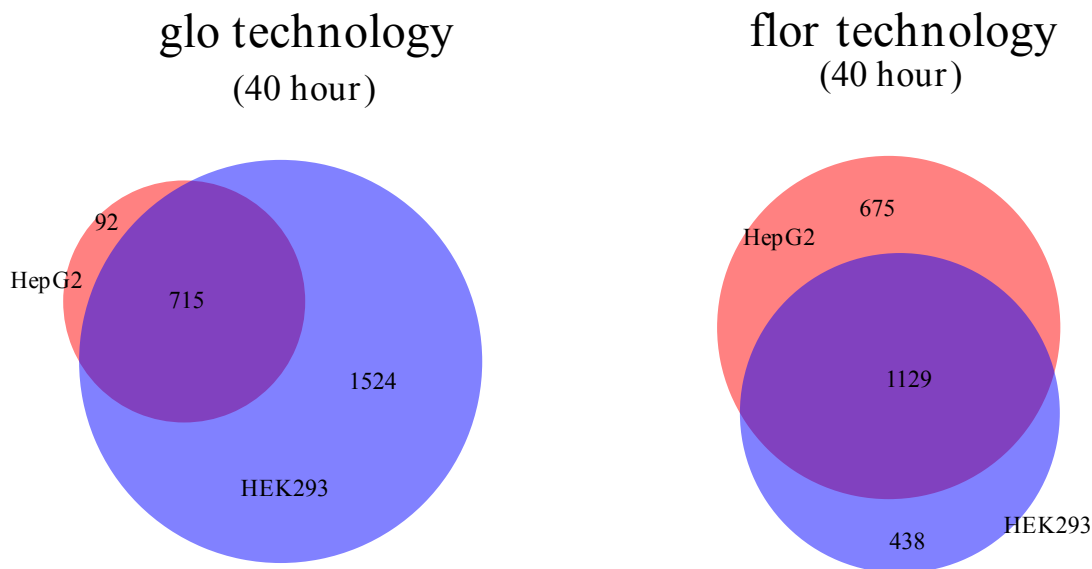

b)

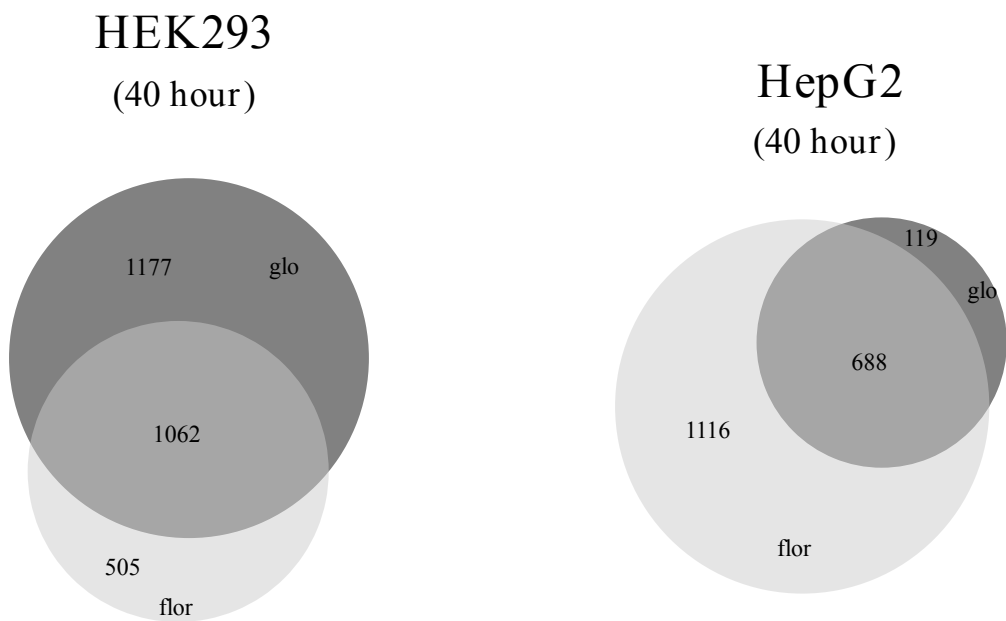

Supplement: S2 Fig — a) comparison between cell lines using either glo or flor technology. b) comparison between assay technologies using either HEK293 or HepG2 cell line. (PDF) [file pone.0177902.s002.pdf]

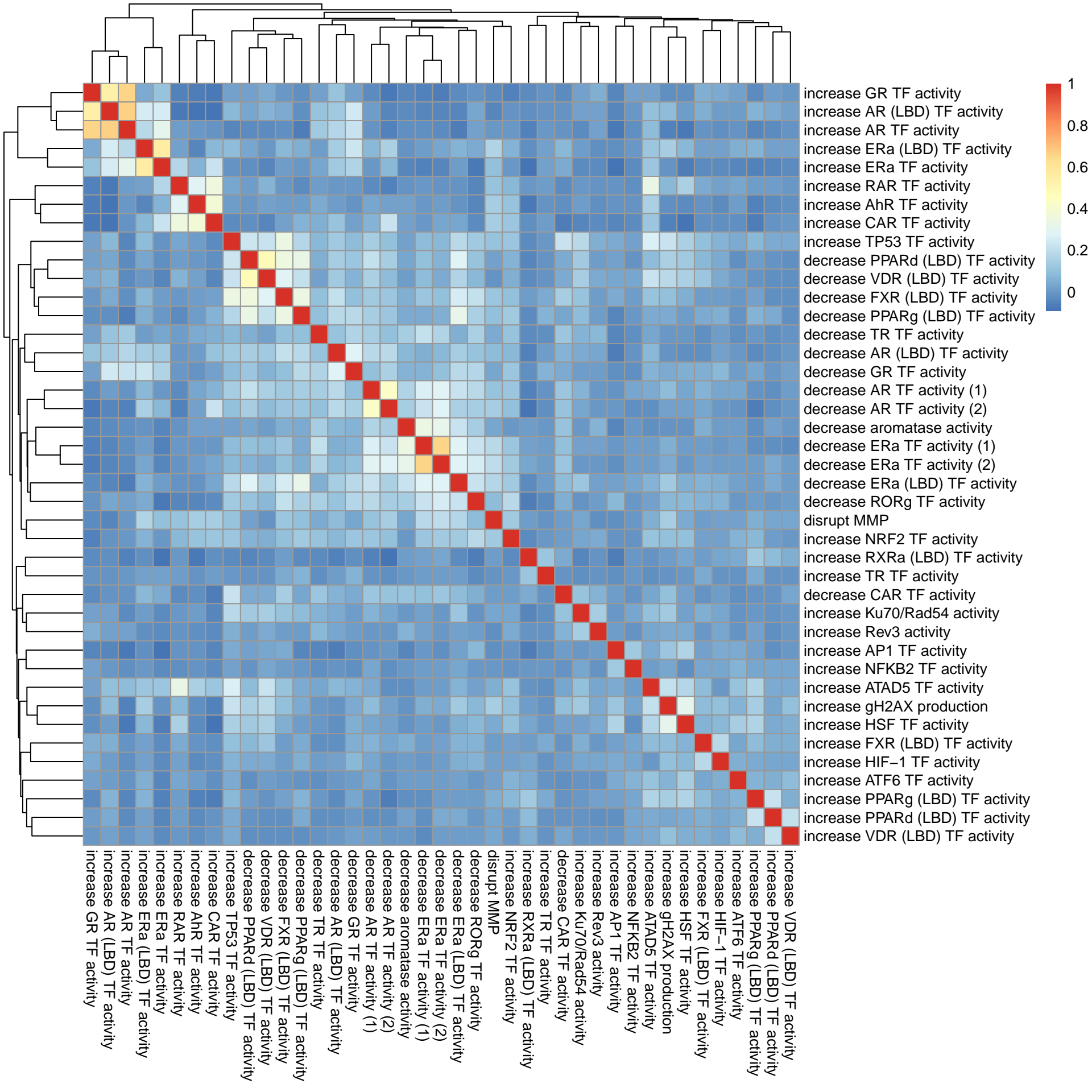

Supplement: S3 Fig — The log10(point-of-departure (POD)) activity value was used when comparing pathways. The 1 molar concentration activity value was set for the inactive and inconclusive chemicals (artifacts included). Pearson’s correlation between toxicity pathways was calculated. Only chemicals active in at least one of the pathways were included. The average linkage was used to connect the pathways with similar activity profile. (PDF) [file pone.0177902.s003.pdf]

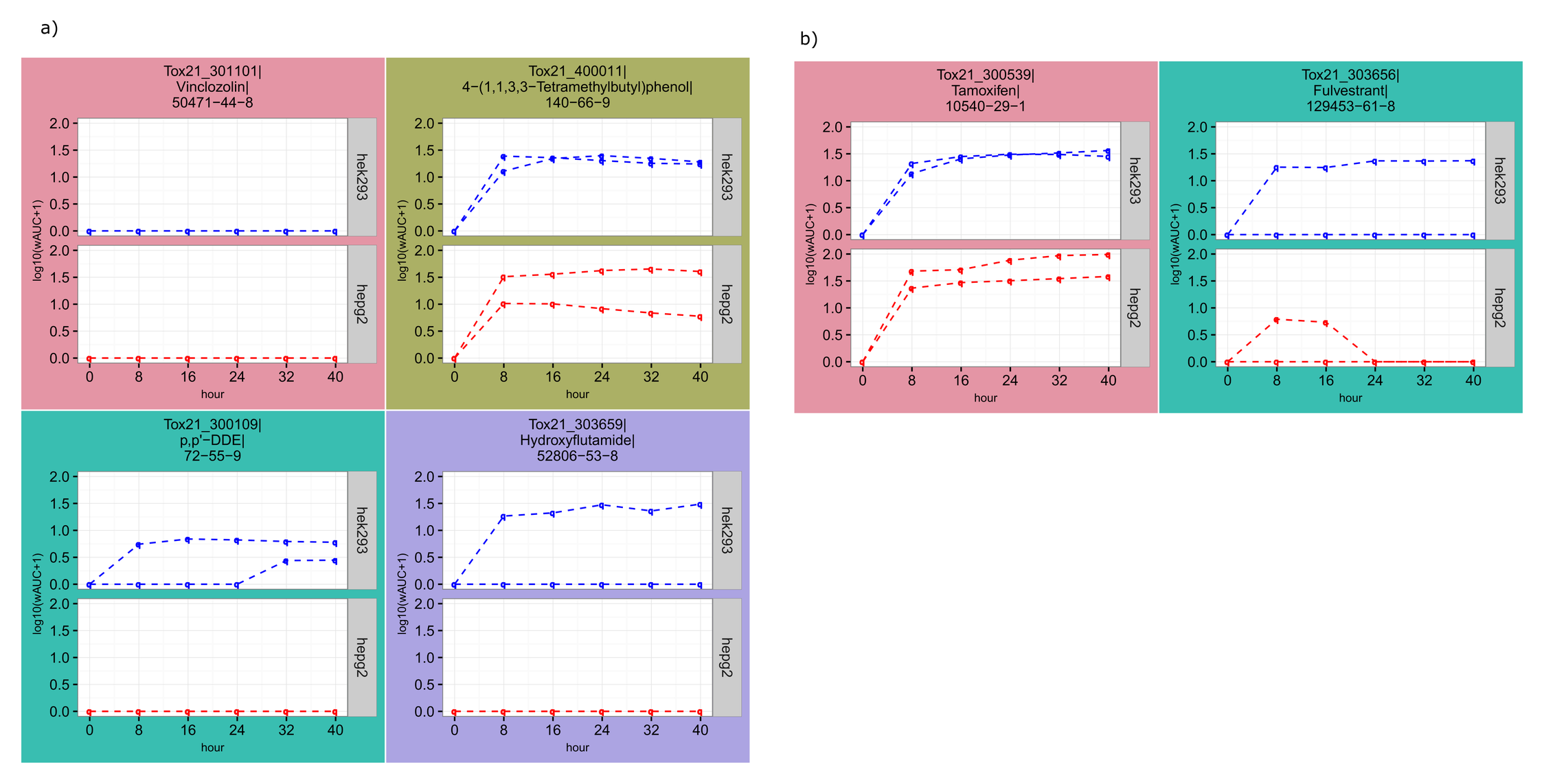

Supplement: S4 Fig — a) AR. The background color corresponds to the clusters presented in Fig 5A; filled circle (glo); hollow circle (flor). b) ER. The background color corresponds to the clusters presented in Fig 5B; filled circle (glo); hollow circle (flor). (TIF) [file pone.0177902.s004.tif]

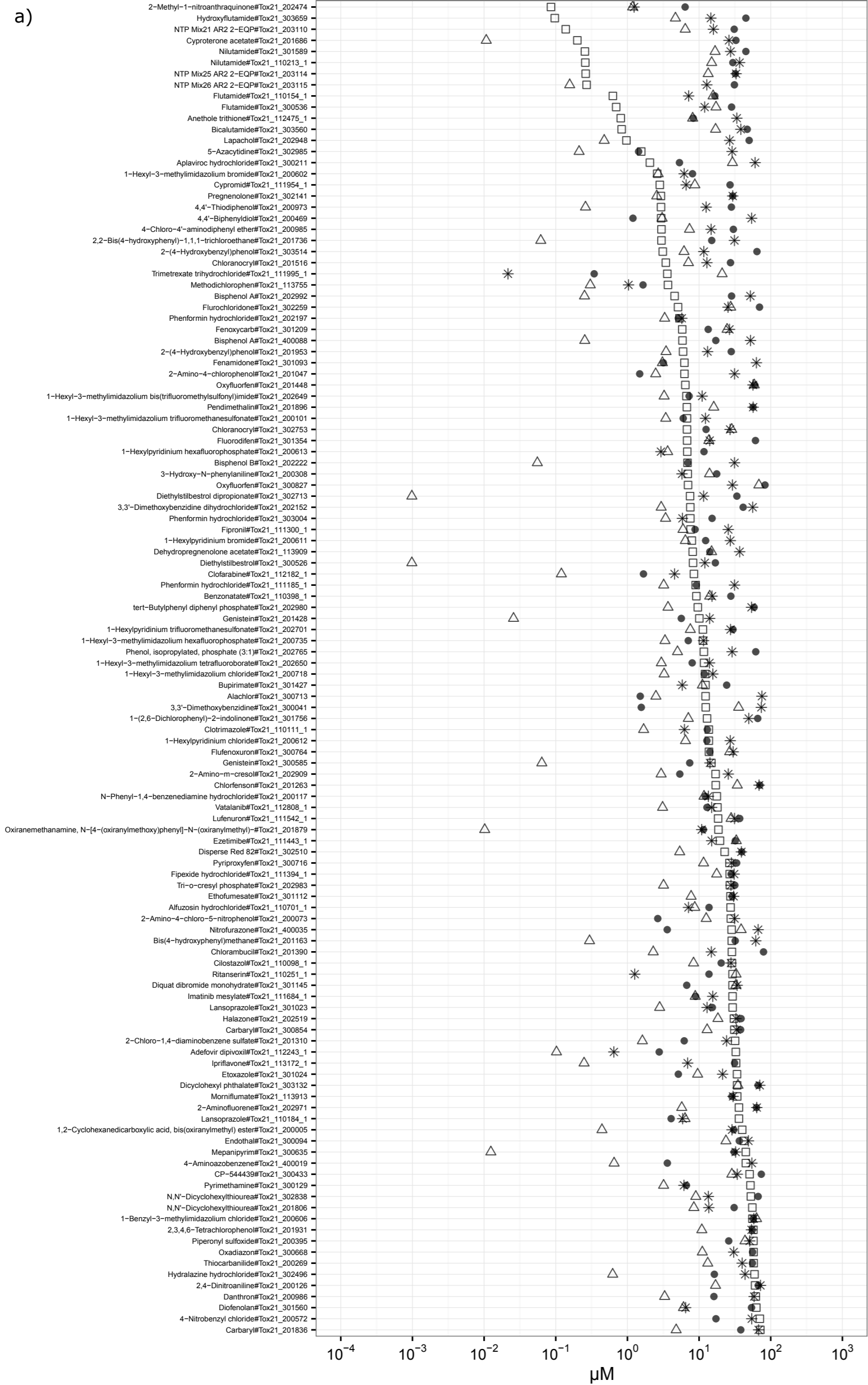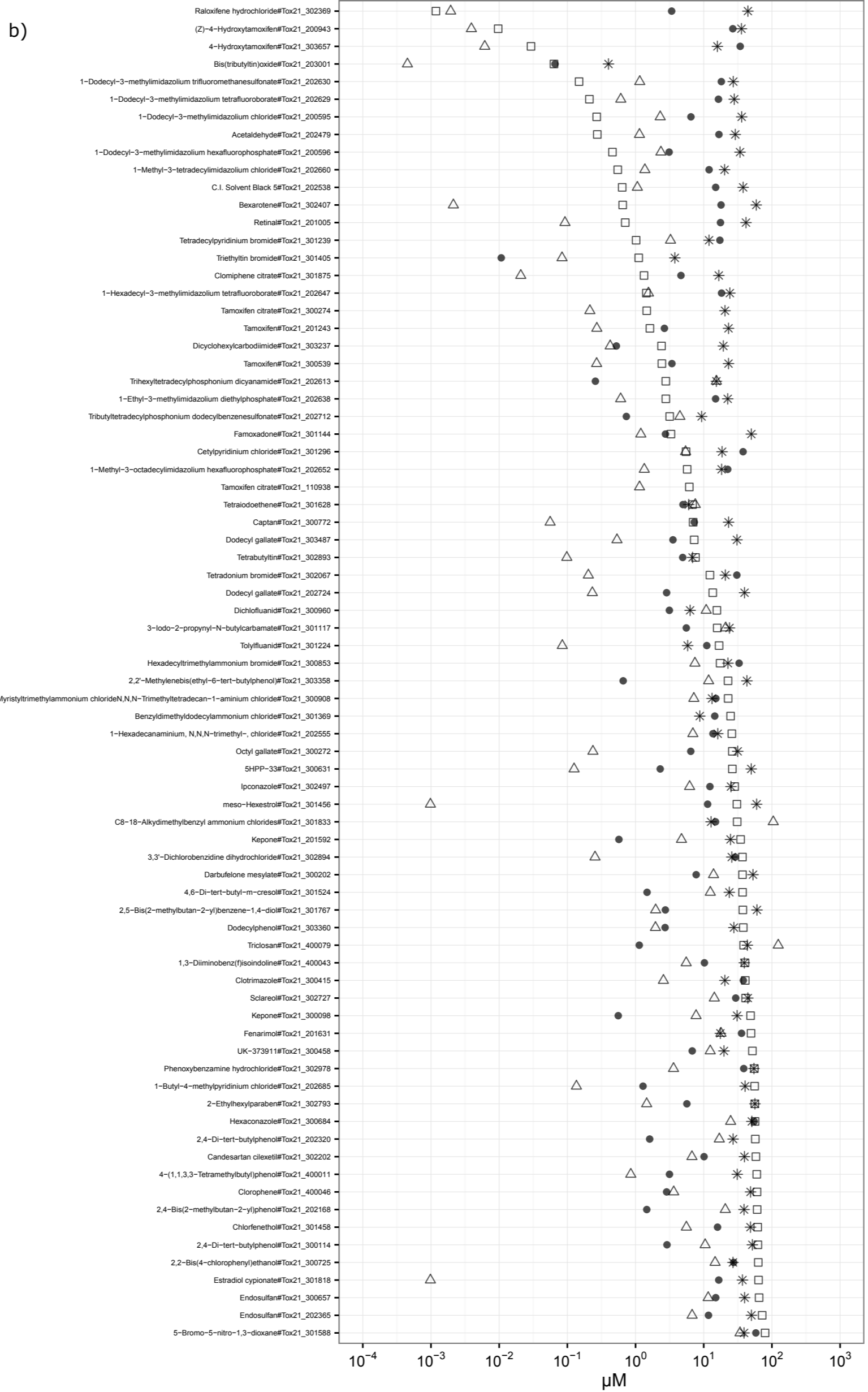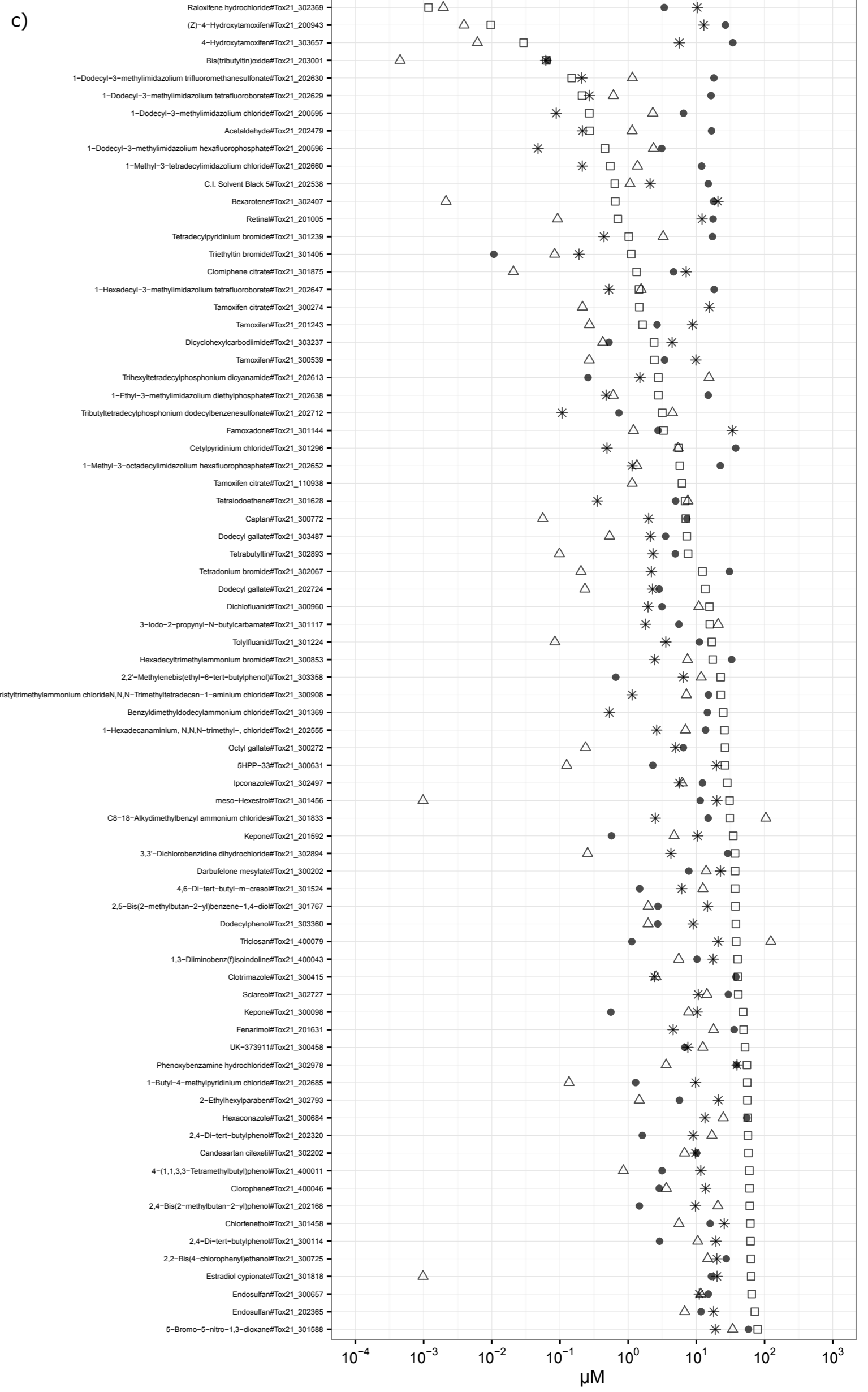

Supplement: S5 Fig — a) chemicals that decrease AR TF activities in the any of the three Tox21 assays (tox21-ar-bla-antagonist-p1, tox21-ar-mda-kb2-luc-antagonist-p1, tox21-ar-mda-kb2-luc-antagonist-p2, see Table A in S1 Text for the description). b) chemicals that decrease ER TF activities in any of the two Tox21 assays (tox21-er-luc-bg1-4e2-antagonist-p1, tox21-er-luc-bg1-4e2-antagonist-p2). Median activity in real-time cytotoxicity assays was used. c) chemicals that decrease ER TF activities in any of the two Tox21 assays (tox21-er-luc-bg1-4e2-antagonist-p1, tox21-er-luc-bg1-4e2-antagonist-p2). Most potent activity in real-time cytotoxicity assays was used. (PDF) [file pone.0177902.s005.pdf]
