# Supplementary material for: Real-time cell toxicity profiling of Tox21 10K compounds reveals cytotoxicity dependent toxicity pathway linkage
Source: PLoS One. 2017 May 22;12(5):e0177902. doi: 10.1371/journal.pone.0177902 (PMC5439695; doi:10.1371/journal.pone.0177902)
Supplement: S1 Text — Table A. Tox21 assays used in the enrichment analysis. Public data can be downloaded from https://tripod.nih.gov/tox21/assays/. Table B. Assay performance evaluation. Table C. Number of actives at each time point and the fold change of actives between two consecutive time points. (DOCX) [file pone.0177902.s006.docx]

# Supporting Information

# Tables

## Table A. Tox21 assays used in the enrichment analysis.

| database protocol name | protocol name | cell type | species | time point of measurement (hrs) | PubChem AID  (Summary) |
| --- | --- | --- | --- | --- | --- |
| Nuclear receptor related | | | | | |
| tox21-ahr-p1 | increase AhR TF activity | HepG2 | human | 24 | 743122 |
| tox21-ar-bla-agonist-p1 | increase AR (LBD) TF activity | Hek293 | human | 16 | 743053 |
| tox21-ar-bla-antagonist-p1 | decrease AR (LBD) TF activity | Hek293 | human | 18 | 743063 |
| tox21-ar-mda-kb2-luc-agonist-p1 | increase AR TF activity | MDA-kb2 | human | 16 | 743040 |
| tox21-ar-mda-kb2-luc-antagonist-p1 | decrease AR TF activity (1) | MDA-kb2 | human | 16 | 743054 |
| tox21-ar-mda-kb2-luc-antagonist-p2 | decrease AR TF activity (2) | MDA-kb2 | human | 16 | N/A |
| tox21-aromatase-p1 | decrease aromatase activity | MCF-7 | human | 24 | 743139 |
| tox21-car-agonist-p1 | increase CAR TF activity | HepG2-hCAR1-CYP2B6 | human | 24 | 1224839  (Confirmatory) |
| tox21-car-antagonist-p1 | decrease CAR TF activity | HepG2-hCAR1-CYP2B6 | human | 24 | 1224838  (Confirmatory) |
| tox21-er-bla-agonist-p2 | increase ERa (LBD) TF activity | Hek293 | human | 16 | 743077 |
| tox21-er-bla-antagonist-p1 | decrease ERa (LBD) TF activity | Hek293 | human | 22 | 743078 |
| tox21-er-luc-bg1-4e2-agonist-p2 | increase ERa TF activity | BG1 | human | 22 | 743079 |
| tox21-er-luc-bg1-4e2-antagonist-p1 | decrease ERa TF activity (1) | BG1 | human | 22 | 743091 |
| tox21-er-luc-bg1-4e2-antagonist-p2 | decrease ERa TF activity (2) | BG1 | human | 22 | N/A |
| tox21-fxr-bla-agonist-p2 | increase FXR (LBD) TF activity | Hek293 | human | 16 | 743239 |
| tox21-fxr-bla-antagonist-p1 | decrease FXR (LBD) TF activity | Hek293 | human | 16 | 743240 |
| tox21-gh3-tre-agonist-p1 | increase TR TF activity | GH3 | rat | 24 | 743066 |
| tox21-gh3-tre-antagonist-p1 | decrease TR TF activity | GH3 | rat | 24 | 743067 |
| tox21-gr-hela-bla-agonist-p1 | increase GR TF activity | HeLa | human | 16 | 720719 |
| tox21-gr-hela-bla-antagonist-p1 | decrease GR TF activity | HeLa | human | 16 | 720725 |
| tox21-ppard-bla-agonist-p1 | increase PPARd (LBD) TF activity | Hek293 | human | 16 | 743227 |
| tox21-ppard-bla-antagonist-p1 | decrease PPARd (LBD) TF activity | Hek293 | human | 16 | 743226 |
| tox21-pparg-bla-agonist-p1 | increase PPARg (LBD) TF activity | Hek293 | human | 16 | 743140 |
| tox21-pparg-bla-antagonist-p1 | decrease PPARg (LBD) TF activity | Hek293 | human | 16 | 743199 |
| tox21-rar-agonist-p1 | increase RAR TF activity | C3H10T1/2 | mouse | 6 | 1159553 |
| tox21-ror-cho-antagonist-p1 | decrease RORg TF activity | CHO | chicken | 18 | 1159523 |
| tox21-rxr-bla-agonist-p1 | increase RXRa (LBD) TF activity | Hek293 | human | 16 | 1159531 |
| tox21-vdr-bla-agonist-p1 | increase VDR (LBD) TF activity | Hek293 | human | 16 | 743241 |
| tox21-vdr-bla-antagonist-p1 | decrease VDR (LBD) TF activity | Hek293 | human | 16 | 743242 |
| Stress response | | | | | |
| tox21-ap1-agonist-p1 | increase AP1 TF activity | ME-180 | human | 5 | 1159528 |
| tox21-are-bla-p1 | increase NRF2 TF activity | HepG2 | human | 16 | 743219 |
| tox21-dt40-dsb-p1 | increase Ku70/Rad54 activity | DT40 | chicken | 40 | N/A |
| tox21-dt40-srf-p1 | increase Rev3 activity | DT40 | chicken | 40 | N/A |
| tox21-elg1-luc-agonist-p1 | increase ATAD5 TF activity | Hek293 | human | 16 | 720516 |
| tox21-esre-bla-p1 | increase ATF6 TF activity | HeLa | human | 5 | 1159519 |
| tox21-h2ax-cho-p2 | increase gH2AX production | CHO-K1 | hamster | 3 | 1224845  (Confirmatory) |
| tox21-hre-bla-agonist-p1 | increase HIF-1 TF activity | ME-180 | human | 5 | 1224846  (Confirmatory) |
| tox21-hse-bla-p1 | increase HSF TF activity | HeLa | human | 5 | 743228 |
| tox21-mitotox-p1 | disrupt MMP | HepG2 | human | 1 | 720637 |
| tox21-nfkb-bla-agonist-p1 | increase NFKB2 TF activity | ME-180 | human | 5 | 1159518 |
| tox21-p53-bla-p1 | increase TP53 TF activity | HCT-116 | human | 16 | 720552 |

Public data can be downloaded from <https://tripod.nih.gov/tox21/assays/>. Abbreviations: TF: transcriptional factor; LBD: ligand binding domain; AhR: aryl hydrocarbon receptor; AR: androgen receptor; CAR: constitutive androstane receptor; ERα: estrogen receptor alpha; FXR: farnesoid X receptor; GR: glucocorticoid receptor; PPARδ: peroxisome proliferator-activated receptor delta; PPARγ: peroxisome proliferator-activated receptor gamma; RAR: retinoic acid receptor; RXR: retinoid X receptor; VDR: vitamin D receptor; MMP: mitochondrial membrane potential; RORγ: RAR-related orphan receptor gamma; N/A: not released. Some assays have been used to screen Tox21 library twice with different experimental protocols.

## Table B. Assay performance evaluation.

| **RealTime-Glo™ MT Cell Viability Assay in HEK293 (n=136)** | **Time course (hrs)** | **8** | **16** | **24** | **32** | **40** |
| --- | --- | --- | --- | --- | --- | --- |
|  | IC_50_ (μM) | 5.91±1.26 | 2.53±0.74 | 1.34±0.52 | 0.87±0.44 | 0.79±0.32 |
|  | CV% (DMSO) | 6.96±1.55 | 6.68±1.47 | 6.38±1.53 | 6.27±1.46 | 6.19±1.50 |
|  | Z’ | 0.73±0.05 | 0.74±0.05 | 0.76±0.05 | 0.77±0.05 | 0.78±0.05 |
|  | S/B | 10.84±0.98 | 12.35±1.38 | 14.06±1.61 | 15.28±2.55 | 16.97±1.95 |
|  |  |  |  |  |  |  |
| **CellTox Green Cytotoxicity Assay in HEK293 (n=136)** | **Time course (hrs)** | **8** | **16** | **24** | **32** | **40** |
|  | EC_50_ (μM) | 9.88±2.10 | 8.01±1.52 | 6.06±1.11 | 6.73±1.62 | 7.69±1.76 |
|  | CV% (DMSO) | 6.86±1.40 | 7.56±1.49 | 7.95±1.51 | 8.12±1.55 | 8.22±1.55 |
|  | Z’ | 0.75±0.04 | 0.78±0.03 | 0.72±0.04 | 0.70±0.05 | 0.79±0.03 |
|  | S/B | 3.56±0.12 | 3.29±0.12 | 3.42±0.17 | 4.18±0.59 | 5.25±0.29 |
|  |  |  |  |  |  |  |
| **RealTime-Glo™ MT Cell Viability Assay in HepG2 (n=136)** | **Time course (hrs)** | **8** | **16** | **24** | **32** | **40** |
|  | IC_50_ (μM) | 7.90±1.62 | 7.53±1.44 | 7.79±1.54 | 8.05±1.68 | 8.18±1.91 |
|  | CV% (DMSO) | 3.31±0.81 | 2.60±0.62 | 2.24±0.72 | 2.47±1.83 | 2.86±1.32 |
|  | Z’ | 0.87±0.02 | 0.90±0.02 | 0.91±0.02 | 0.90±0.05 | 0.89±0.04 |
|  | S/B | 26.14±1.02 | 27.18±1.02 | 27.56±1.11 | 27.15±4.15 | 27.53±1.54 |
|  |  |  |  |  |  |  |
| **CellTox Green Cytotoxicity Assay in HepG2 (n=136)** | **Time course (hrs)** | **8** | **16** | **24** | **32** | **40** |
|  | EC_50_ (μM) | 7.59±1.48 | 6.18±1.27 | 5.87±1.10 | 6.43±1.00 | 6.40±0.96 |
|  | CV% (DMSO) | 8.26±2.24 | 8.79±3.57 | 9.58±5.50 | 9.67±6.79 | 9.83±7.08 |
|  | Z’ | 0.73±0.07 | 0.72±0.09 | 0.71±0.10 | 0.78±0.08 | 0.78±0.10 |
|  | S/B | 3.69±0.38 | 3.61±0.54 | 4.42±0.79 | 5.50±1.25 | 6.08±0.94 |

S/B: signal to background ratio, marginal ([2,3]), excellent (>3); CV(%): covariance of raw reads, acceptable (< 10%); Z’: Z-factor, marginal ([0,0.5]), excellent (>0.5); EC_50_/IC_50_: half maximal effect or inhibition concentration; data not available for 0 time point as the exposure duration was not long enough for the positive control to have an effect.

## Table C. Number of actives in each time point and the fold change of actives between two consecutive time points

| hour  protocol | | 0 | 8 | 16 | 24 | 32 | 40 |
| --- | --- | --- | --- | --- | --- | --- | --- |
| hek293_flor | # of actives | 156 | 760 | 868 | 1163 | 1370 | 1567 |
|  | # of active at x + 8/# of active at x | 4.87 | 1.14 | 1.34 | 1.18 | 1.14 | NA |
| hek293_glo | # of actives | 416 | 1669 | 1989 | 2123 | 2206 | 2239 |
|  | # of active at x + 8/# of active at x | 4.01 | 1.19 | 1.07 | 1.04 | 1.01 | NA |
| hepg2_flor | # of actives | 217 | 754 | 1045 | 1434 | 1653 | 1804 |
|  | # of active at x + 8/# of active at x | 3.47 | 1.39 | 1.37 | 1.15 | 1.09 | NA |
| hepg2_glo | # of actives | 550 | 880 | 889 | 922 | 871 | 807 |
|  | # of active at x + 8/# of active at x | 1.60 | 1.01 | 1.04 | 0.94 | 0.93 | NA |

x: 0, 8, 16, 24, 32; NA: not available
